# Advances, challenges and future directions for stem cell therapy in amyotrophic lateral sclerosis

**DOI:** 10.1186/s13024-017-0227-3

**Published:** 2017-11-13

**Authors:** Yuri Ciervo, Ke Ning, Xu Jun, Pamela J. Shaw, Richard J. Mead

**Affiliations:** 1 Sheffield Institute for Translational Neuroscience (SITraN), Department of Neuroscience, Faculty of Medicine, Dentistry and Health, University of Sheffield, 385a Glossop Rd S10 2HQ, Sheffield, UK; 2 Tongji University School of Medicine, 1239 Siping Rd, Yangpu Qu, Shanghai, China

**Keywords:** Neurodegeneration, Amyotrophic lateral sclerosis, Stem cell transplantation, Adipose derived stem cells

## Abstract

Amyotrophic lateral sclerosis (ALS) is a rapidly progressive neurodegenerative condition where loss of motor neurons within the brain and spinal cord leads to muscle atrophy, weakness, paralysis and ultimately death within 3–5 years from onset of symptoms. The specific molecular mechanisms underlying the disease pathology are not fully understood and neuroprotective treatment options are minimally effective.

In recent years, stem cell transplantation as a new therapy for ALS patients has been extensively investigated, becoming an intense and debated field of study. In several preclinical studies using the SOD1^G93A^ mouse model of ALS, stem cells were demonstrated to be neuroprotective, effectively delayed disease onset and extended survival. Despite substantial improvements in stem cell technology and promising results in preclinical studies, several questions still remain unanswered, such as the identification of the most suitable and beneficial cell source, cell dose, route of delivery and therapeutic mechanisms. This review will cover publications in this field and comprehensively discuss advances, challenges and future direction regarding the therapeutic potential of stem cells in ALS, with a focus on mesenchymal stem cells. In summary, given their high proliferation activity, immunomodulation, multi-differentiation potential, and the capacity to secrete neuroprotective factors, adult mesenchymal stem cells represent a promising candidate for clinical translation. However, technical hurdles such as optimal dose, differentiation state, route of administration, and the underlying potential therapeutic mechanisms still need to be assessed.

## Background

Amyotrophic lateral sclerosis (ALS), also known as Lou Gehrig’s disease, is a rapidly progressive neurodegenerative condition characterized by selective degeneration of both upper motor neurons (MNs) in the motor cortex, and lower motor neurons in the brainstem and ventral horn of the spinal cord [1]. The estimated incidence of ALS across the world is 2/100,000, with a prevalence of up to 7.4/100000 [2].

The disease typically manifests during the sixth to seventh decade of life leading to progressive muscle atrophy, weakness and paralysis. Affected individuals usually die within 2 to 5 years after diagnosis due to respiratory failure [2]. ALS is mainly sporadic in origin (SALS) but a family history of the disorder can be found in ~10% of cases. Hereditary forms of the disease (familial ALS or FALS), are predominantly autosomal dominant and rarely X-linked or recessive [2].

More than 20 mutated genes have been found to cause FALS including SOD1 [3], TARDBP [4, 5], FUS [6, 7], OPTN [8], VCP [9, 10], UBQLN2 [11], C9orf72 [12, 13] and very recently TBK1 [14, 15]. SALS and FALS are clinically indistinguishable, and since mutations in FALS genes are also present in sporadic or isolated cases of ALS, the disease can be interpreted as complex and multi-factorial [16]. Nevertheless, clinical variability such as rate of progression, site of onset (limb or bulbar) and survival within patients and even relatives who carry the same gene mutation highlight the importance of external factors which may play a role in the susceptibility and age of onset of the disease [16].

The specific molecular mechanisms behind ALS onset, development and progression are not fully understood. However, the discovery of causative inherited and de novo gene mutations, together with the generation of the SOD1^G93A^ transgenic mouse model uncovered important pathological mechanisms in ALS [17, 18]. The SOD1^G93A^ mice demonstrate many of the features seen in human ALS pathology and represent the most widely used in vivo model for the study of ALS. Indeed, axon retraction, selective spinal motor neuron death, loss of innervation of motor end-plates, muscular atrophy and progressive motor deficit with terminal paralysis of hind limbs are observed in this murine model [17, 18].

**Fig. 1:**
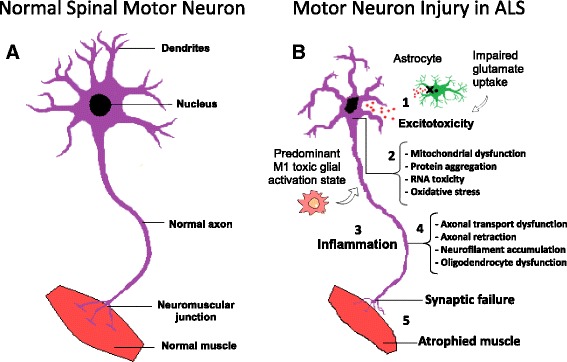
Molecular mechanisms in the pathology of amyotrophic lateral sclerosis. **a** Schematic representation of healthy spinal cord motor neuron. **b** Schematic representation of ALS affected spinal cord motor neuron: 1) Astrocytes are not able to support neuronal functions and impaired glutamate clearance leads to neuronal excitotoxicity; 2) Defects in protein degradation pathways and disturbances in RNA processing result in protein aggregate formation, RNA toxicity and mitochondrial dysfunction; 3) The secretion of pro-inflammatory cytokines by predominant M1 activated microglia contributes to the development of an inflammatory milieu; 4) Failure of axonal architecture and transport functions, together with the alteration of the physiological role of oligodendrocytes results in 5) synaptic failure, denervation and finally, muscle atrophy

Several pathophysiological mechanisms have been proposed including: cytoplasmic protein mis-localization and aggregation [19], aberrant protein homeostasis [20], RNA toxicity [13], dysregulation of RNA processing [21], excitotoxicity mediated by excessive glutamate receptor activation [22], mitochondrial dysfunction [23], endoplasmic reticulum stress response and microglial activation [24], abnormal rearrangement of the cytoskeleton with impaired axonal transport [25], and oxidative stress [26]. Moreover, the contribution of microglial cells, oligodendrocytes and astrocytes seems to be critical for the development of the disease influencing significantly the speed of disease progression after onset [27–31]. Indeed, ALS is considered as a non-cell autonomous disease, where the start and progression of motor neuron degeneration seems to be influenced by complex interaction among different kinds of cells, together with the development of a sustained inflammatory milieu [32]. Figure 1 summarizes the major pathological mechanisms contributing to motor neuron injury in ALS.

The complex heterogeneity of ALS, where several molecular mechanisms contribute to the pathology, enables various opportunities for therapeutic intervention. However, the complexity of the disease and clinical variability within patients inevitably makes the identification of a universal single drug or therapy capable of correcting the pathophysiology of ALS in its totality, very difficult. Various therapeutic strategies are being experimentally evaluated such as immunomodulation, approaches to improve mitochondrial function, induction of autophagy and anti-oxidant agents [16]. However, after over twenty years of encouraging results in preclinical studies, no efficacious treatment has been developed so far and riluzole, the only recognised neuroprotective agent in ALS, prolongs life expectancy by only approximately 3–4 months [16].

During the last decade, progress in stem cell biology has paved the way for potential cellular based therapy in neurological diseases. Albeit still at an early stage and with several issues to be solved, stem cell therapy holds great promise for the treatment of ALS. Stem cells are a population of cells which are defined by functional characteristics. They are undifferentiated cells capable of self-renewal, able to form clones in vitro and capable of differentiation into mature cell lines of various tissues.

There are several potential advantages to the use of stem cells in ALS:

1) The complexity of ALS pathology may not allow the use of a single drug or targeted treatment;

2) The capability of stem cells to differentiate into neuron-like cells and potentially replace the neuronal population lost in ALS;

3) The degeneration of existing motor neurons could be prevented by the release of neuroprotective trophic factors and the immunomodulatory properties of transplanted stem cells, thus modifying the toxic microenvironment in ALS.

This review will outline the recent progress relevant to stem cell-based therapies in general in ALS, and will focus on the therapeutic potential of mesenchymal stem cells by discussing the major technical issues, challenges and future directions.

## Stem cell therapy in ALS

There are different types of stem cells which differ according to the source, clonogenic capacity, differentiation potential and availability.

### Human embryonic stem cells (hESC)

Human embryonic stem cells (hESC) are derived from the inner cell mass of the blastocyst and can indefinitely propagate in vitro*,* preserving the capacity to differentiate into any cell type of the three embryonic germ layers (endoderm, mesoderm and ectoderm) [33]. For the first time in 2005, Shin and colleagues obtained motor neuron-like cells expressing markers such as islet1 and choline acetyltransferase from hESC using conditioned media containing basic fibroblast growth factor (bFGF), retinoic acid (RA) and sonic hedgehog (Shh) [34]. The survival, differentiation and beneficial neurotrophic support of motor neuron progenitors (MNP) derived from hESC has also been demonstrated after lumbar intraspinal transplantation into SOD1^G93A^ mice and other MND models [35, 36]. Wyatt et al., transplanted hESC derived MNPs directly into the spinal cord of immunosuppressed SOD1^G93A^ mice, spinal muscular atrophy (SMA) Δ7SMN pups and rats with spinal cord injury (SCI), demonstrating the in vivo differentiation of the engrafted cells into a mixed population of mature and immature motor neuron cells [36]. The axons of the differentiated cells did not reach the periphery, and the authors did not prove the integration of the differentiated cells into the existing neural circuit. However, the transplanted cells were able to reduce motor neuron loss in proximity to the injection site by actively releasing neurotrophic factors such as neurotrophin-3 (NT-3) and nerve growth factor (NGF) [36]. In particular, in SOD1^G93A^ mice that received MNPs, 43 ± 5 endogenous neurons cranial to the injection site survived until the end of the study (110 days old), in comparison to the vehicle control group in which 27 ± 3 neurons were counted [36]. Yet, the use of hESCs in the clinic is hindered because of ethical concerns, potential tumorigenicity in vivo and the potential for graft rejection [37].

### Foetal neural progenitors (NSC)

Foetal neural progenitors (NSC) are multipotent stem cells derived from foetal spinal cord or brain, capable of in vitro self-renewal and able to differentiate into astrocytes, neurons and oligodendrocytes. Given their partial maturation state they have less propensity to form teratomas in vivo [38]. Several studies investigated the safety and therapeutic potential of spinal, intrathecal or intracranial transplantation of hNSC in ALS rodent models [39–41]. In particular, a well-characterized hNSC cell line (NSI-566RSC) derived from an 8-week human foetal spinal cord showed very promising results in transplanted SOD1^G93A^ rodents [42, 43].

In 2006, Yan et al. performed spinal cord injections of NSI-566RSC cells in the ventral horn of 8-week-old SOD1^G93A^ mice at the lumbar level L4-L5, under combined immunosuppression or CD4 antibodies [42]. Four separate injections were carried out per mouse, with a total of 8 × 10^4^ cells. The authors showed that the graft survived for more than two months after transplantation, with most of the engrafted NSCs showing differentiation into TUJ1^+^ neurons, and evidence of synaptic contacts with host neurons [42]. Moreover, in mice injected with live NSCs cells, disease onset was delayed by 15 days and life span extended by 12 days in comparison to the control group that received injections of dead cells. A statistically significant later onset and a slowing of disease progression, was also confirmed by analysis of motor performance [42]. The same group of authors, investigated the therapeutic potential of the NSCs-566RSC cell line after injection of around 8 × 10^5^ cells into the lumbar spinal cord of SOD1^G93A^ rats at a pre-symptomatic disease stage [43]. In this study, rats that received live NSCs showed an increase in survival of around 11 days and a delay in disease onset of 7 days when compared to the control placebo group. The beneficial effect could be associated with the release of neurotrophins such as glial-derived neurotrophic factor (GDNF) and brain-derived neurotrophic factor (BDNF), which in turn delayed the death of α-motor neurons in the lumbar region [43].

Despite these encouraging data, the restricted number of cells available for transplantation represents a potential limitation for obtaining therapeutic efficacy in humans.

### Induced pluripotent stem cells (iPSCs)

Induced pluripotent stem cells (iPSC) are an adult source of pluripotent stem cells derived from somatic cells (e.g. dermal fibroblasts) by forced genetic induction of four factors that preserve pluripotency in ESCs (KLF4, SOX2, OCT4 and c-MYC) [44, 45]. The differentiation of human iPSCs into electrically active motor neurons has been accomplished by several groups both in vitro and in vivo [46, 47].

Interestingly, Popescu et al. demonstrated in vivo differentiation of human iPSC-derived neural progenitors (NPs) in presymptomatic SOD1^G93A^ rats following stem cell injection into the ventral horns of the lumbar spinal cord [48]. At 30 days post-transplantation human mitochondria positive cells displayed expression of the neuronal precursor marker doublecortin (DCX), indicating the presence of undifferentiated progenitors. Substantial differentiation into mature motor neurons could be observed only after 60 days, with the majority of engrafted cells expressing the neuronal marker MAP2 [48]. This relatively long time is something to bear in mind, considering that transplantation was performed before disease onset and NPs could survive and differentiate within a less toxic environment in comparison to the symptomatic stage. If cells were transplanted during disease progression, as would occur in the clinic, NPs may not have a permissive environment and/or the necessary time to differentiate into mature motor neurons. The therapeutic potential of iPSC-derived NPs has also been investigated in the SOD1^G93A^ mouse model of ALS [49]. iPSC-derived neural stem cells (NSCs), further selected for the expression of the integrin VLA4^+^, were transplanted either by repeated (*n* = 3) intrathecal or weekly intravenous injections. Intrathecal injection of cells extended survival by 10 days, while systemic delivery increased survival by 23 days, compared to control PBS injected mice [49]. The molecular protective mechanism of iPSC-derived NSCs may be attributed to the capacity of these cells to secrete trophic factors such as GDNF, BDNF, NT-3 and TGF-α, which in turn protects resident motor neurons and reduces astrogliosis [49].

The opportunity for reprogramming somatic cells into neural stem cells (NSCs) could overcome the immune rejection problem by autologous transplantation, and bypass the ethical problems related to the use of ESCs and foetal cells. However, several issues need to be addressed, such as reprogramming efficiency, epigenetic memory and safety before translation of the use of iPSCs into clinical practice.

### Mesenchymal stem cells (MSCs)

Adult mesenchymal stem cells (MSCs) are stromal multipotent stem cells that can be derived from umbilical cord, bone marrow, adipose tissue and peripheral blood and have the capacity to differentiate into different components of mesodermal origin (cartilage, bone, fat, muscle and stroma) [50]. These cells can be expanded and maintained by several passages in plastic-adherent tissue culture. MSCs show fibroblast-like morphology, they do not differentiate spontaneously and are characterized by the expression of specific surface markers [50]. In addition to mesodermal commitment, several authors showed the potential of bone marrow-derived MSCs (BM-MSC) to differentiate into neuron-like cells, oligodendrocytes and astrocytes [51–59].

Several characteristics make the use of MSCs very attractive in ALS cell therapy. MSCs can be obtained and expanded from adults relatively easily, bypassing the ethical constraints related to the use of embryonic and human foetal derived stem cells. Also, they are less immunogenic and can be harvested from ALS patients so allowing both allogenic and autologous transplantation [50]. Moreover, these cells are capable of homing to areas of insult, possess immunomodulation and anti-apoptotic properties, and are capable of secreting several cytokines, extracellular matrix proteins and growth factors relevant in neuroprotection and tissue repair [60]. Because of these properties, mesenchymal stem cells are receiving significant attention amongst researchers.

## Proof-of-concept for MSCs therapy in the SOD1^G93A^ model of ALS

Several studies were performed in ALS rodent models in order to investigate the potential of either human (hMSC) or murine (mMSC) bone marrow-derived MSCs (BM-MSCs) for cell therapy in ALS. Different approaches have been tested by varying the delivery method, the amount of injected cells, the timing of intervention, and the differentiation state. Table 1 shows a summary of preclinical studies described in the literature with injection of BM-MSCs in ALS models.

**Table 1:** Summary of BM-MSC injections in ALS rodent model

ALS model		Delivery Method	Cell numbers	Age	Sacrifice to evaluate graft	Outcomes	Cell graft	Reference
SOD1 ^G93A^ mice	hBM-MSC after 5 passages in culture	Intravenous	3 × 10 ^6^ in 0.3 ml of L-DMEM	Pre-symptomatic (8 w)	14 days post- injection	Increased lifespan of 18 days, delayed disease onset of 14 days and reduced motor neuron loss	Very few cells in grey and white matter of lumbar spinal cord. Considerable number of cells in kidney, lung and spleen.	[61]
SOD1 ^G93A^ mice	hBM-MSC expressing Ngn1 after 5 passages in culture	Intravenous	1 × 10 ^6^ in 0.1 ml of PBS	Pre-symptomatic (8 w) or symptom onset (14–16 w)	14 days post- injection	Increased lifespan of 3 days, delayed disease onset of 5 days and reduced motor neuron loss.	Very few cells in brainstem and spinal cord. Cells mostly found in kidney.	[58]
SOD1 ^G93A^ mice	mBM-MSCs expressing Luciferase expanded for 8–15 passages	Intravenous	1 × 10 ^6^ in 0.2 ml of PBS	Symptom onset	24 h, 3 weeks and 4 weeks post-Injection	Increased lifespan of 17 days, delayed decline in motor performance and weight loss.	Cells detected in spinal cord and hypothalamus after 24 h and 48 h. Very few cells after 20 days. No cells after 35 days	[58, 62]
SOD1 ^G93A^ mice	hBM-MSC-derived neural-like cells from neurosphere	Cisterna Magna	1x10 ^5^ in 10 μl of PBS	Pre-symptomatic	10 days post-injection	No benefits	Subarachnoid space near cisterna magna and within cerebellum.	[63]
SOD1 ^G93A^ mice	ALS-hBM-MSC after 3 passages in culture	Cisterna Magna	1 x 10 ^6^ in 10 μl of ALS-CSF	Pre-symptomatic	7 weeks post-injection	Increased lifespan of 8 days, slowed decline in rotarod test and increased motor neuron survival	Ventricular system and subarachnoid space. Some cells into brain and spinal cord.	[64]
SOD1 ^G93A^ mice	hBM-MSC after 3–4 passages in culture	Cisterna Magna	5 × 10 ^5^ in 5 μl of PBS	Pre-symptomatic	3 weeks post-injection	Increased lifespan of 14 days, delayed disease onset of 6 days and reduced astrogliosis	Not shown	[67]
SOD1 ^G93A^ rats	GFP-hBM-MSCs	Cisterna Magna	5x10 ^5^ in 10 μl of PBS	Symptom onset	14 days post-injection	Increased lifespan of 14 days and reduced motor neuron loss. Preservation of PNN.	No graft	[66]
SOD1 ^G93A^ rats	BrdU-labelled mBM-MSC after 15 passages in culture	Cisterna Magna	2 x 10 ^6^ in 15 μl of Opti-MEM	Symptom onset	35 days post-injection	Increased lifespan of 16 days, slowed disease progression, reduced motor neuron loss and inflammation.	White and grey matter of spinal cord. Substantial differentiation into astrocyte phenotype	[54]
SOD1 ^G93A^ mice	Bisbenzimide -hBM-MSC after 3–8 passages in culture	Cisterna lumbaris (L5-L6)	3 × 10 ^5^ in 5 μl of PBS	Symptom onset	14 days post- injection	Reduced astrogliosis and microglial activation.	Lumbar, cervical and thoracic meninges. Migration into spinal cord parenchyma.	[68]
SOD1 ^G93A^ mice	Bisbenzimide -hBM-MSC after 3–5 passages in culture	Intraspinal (L1-L2)	1 × 10 ^5^ in 2 μl of PBS	Pre-symptomatic (28 w)	10 weeks post- injection (38 w)	Reduced astrogliosis and microglial activation. Improved motor function and delayed neuron death	Close to injection site. Migration up to 2 mm toward ventral horn.	[70]
SOD1 ^G93A^ rats	GFP-hBM-MSC engineered to secrete GDNF	Intramuscular after focal injuries	1.3 × 10 ^5^ in	Pre-symptomatic (80 days)	Disease end-point	Prolonged survival, reduction in denervated motor endplates and reduced motor neuron loss	Between basal lamina and muscle fibres	[72]

*hBM-MSC* human bone marrow-derived mesenchymal stem cells, *DMEM* Dulbecco’s modified eagle medium, *Ngn1* neurogenin-1, *PBS* phosphate buffered saline, *mBM-MSC* mouse bone marrow-derived mesenchymal stem cells, *ALS-hBM-MSC* human bone marrow-derived mesenchymal stem cells derived from ALS patient, *ALS-CSF* cerebrospinal fluid derived from ALS patient, *GFP-hBM-MSC* green fluorescent protein labelled hBM-MSC, *PNN* perineural net, *BrdU* bromodeoxyuridine, *GDNF* glial derived neural factor

### Intravenous delivery

In 2007, Zhao and colleagues delivered 3 million hBM-MSCs into 60 day pre-irradiated SOD1^G93A^ mice by intravenous infusion [61]. The recipient mice showed about 14 days delay in disease onset, and prolonged survival of about 18 days in comparison to untreated mice. Moreover, the decrease in motor performance (rotarod test) was delayed by 3 weeks [61]. However, immunostaining experiments showed that only a few transplanted cells migrated and penetrated into the grey and white spinal cord matter, surviving for no more than 20 days [61].

In another study, 1 million engineered hBM-MSCs overexpressing neurogenin1 (Ngn1) were injected in the tail vein of pre-symptomatic SOD1^G93A^ mice [58]. Ngn1 is a transcription factor able to induce neuronal differentiation in MSCs [58]. Two weeks after transplantation, injected cells were found within the brain, spinal cord and liver, and some cells had migrated into the spinal cord parenchyma [58]. The neural induction of hMSCs with neurogenin1 seemed to potentiate the migration capacity and survival of engrafted cells into the central nervous system (CNS) of SOD1^G93A^ mice, resulting in enhanced and prolonged benefits [58]. The migration capacity might be explained by high expression of chemokine receptors such as CCR2 and CXCR4, which were significantly more expressed after neurogenin1 induction. [58]. Interestingly, when MSCs were injected at a pre-symptomatic stage, disease onset in the treated group was delayed by 5 days with an increase in life span of only 3 days. Conversely, when injected close to disease onset, Ng1-MSCs were able to increase survival by about 7 days, suggesting the importance of the time of intervention for stem cell therapy in ALS [58].

Furthermore, the Uccelli group demonstrated that mouse BM-MSCs isolated from non-transgenic mice, were therapeutically effective when transplanted during the symptomatic stage of the disease in the SOD1^G93A^ mice [62]. In this study, mBM-MSCs expanded ex-vivo for 8–15 passages, were transfected with the Luciferase gene reporter vector pL-Luc-HI for in vivo tracking and injected into the tail vein of SOD1^G93A^ mice. The mice that received the MSC transplantation showed an extended survival of 17 days, delayed decline in motor performance and decreased weight loss when compared to the control PBS-injected mice [62]. In addition, the transplantation of MSCs alleviated the pathology of the disease in the ALS spinal cord, by reducing astrogliosis and microglial activation, and by restoring antioxidant components such as glutathione-S-transferase and metallothioneins to their baseline level of expression and activity. However, the engraftment efficiency of intravenous delivered cells within the CNS was very low, with luciferase-positive cells almost completely absent twenty days post- injection. This suggested that MSCs delivered by the intravenous route, can exert clinically positive effects during the disease course that do not correlate with the efficiency of long-term engraftment in the host [62].

### Intrathecal delivery

Using a different approach, several authors have injected a variable number of BM-MSCs directly into the cisterna magna of pre-symptomatic SOD1^G93A^ mice [63–66]. Direct injection into the CSF allows the obstacle of the brain blood barrier (BBB) to be bypassed. Moreover, the injected cells may migrate along the spinal cord possibly reaching segments specifically affected by motor neuron degeneration.

Indeed, it has been demonstrated that the injected BM-MSCs were able to delay disease onset, improve motor performance, ameliorate motor neuron death and prolong survival in transplanted SOD1^G93A^ mice [64–67]. These beneficial effects were enhanced by multiple intrathecal injections or by using a considerable number of MSCs (1 × 10^6^) [64, 65]. However, injected cells rarely migrated into the parenchyma, suggesting a neuroprotective effect of MSCs from the CSF [64, 65, 67]. In fact, it was shown that injection of MSCS into the CSF of SOD1^G93A^ mice consistently inhibited microglial activation and the release of inflammatory molecules, possibly by diffusion of soluble factors where direct contact between cells is not a requirement [67].

In 2009, Boucherie and colleagues, investigated the therapeutic potential of rat wild-type (WT) bromodeoxyuridine-labelled BM-MSCs (BrdU-MSC) by intrathecal injection in SOD1^G93A^ transgenic rats, at disease onset [54]. Stem cell transplantation in SOD1^G93A^ rats resulted in a reduction in the rate of disease progression, as the first signs of paralysis were detected 2 weeks later in in comparison to control mice. Moreover, treated mice showed reduced local inflammatory response and an increase in life span of 16 days [54]. Interestingly, this is the only study to show a significant trans-differentiation of MSCs into astrocyte-like cells in vivo*.* Indeed, transplanted BrdU-MSCs were found to penetrate into the grey matter of the ventral horns, and stained positively for the astrocyte marker GFAP [54]. Remarkably, around 40% of the BrdU-MSCs that successfully migrated in proximity to motor neurons, co-localized with the astrocyte marker, and about 30% of the GFAP-positive cells were actually positive for the BrdU. This study demonstrated a considerable local chimerization of the astroglial population near the site of motor neuron injury in the lumbar spinal cord of SOD1^G93A^ rats [54]. However, cell fusion events with resident astrocytes cannot be excluded. Surprisingly, the overall level of astrogliosis was not changed upon MSCs treatment, and the loss of expression of the astrocyte glutamate reuptake transporter (GLT-1), typically observed in SOD1^G93A^ rats, was not rescued [54].

The perineural net (PNN) is a specialized matrix structure present at a high density around motor neurons and is fundamental in axon development as well as neuronal plasticity [66]. Modification and deterioration of the PNN has been observed in the spinal cord of SOD1^G93A^ rats during neurodegeneration [66]. Interestingly, intrathecal delivery of hBM-MSCs into early post-symptomatic SOD1^G93A^ rats, resulted in partial rescue of PNN structures suggesting a role of MSCs in reactivating CNS plasticity [66]. In this study, stem cell injection increased survival of 14 days in comparison to the placebo group.

Boido et al. (2014) injected a total of 300,000 bisbenzimide pre-labelled hMSCs into the cisterna lumbaris (L5-L6 level) of early symptomatic SOD1^G93A^ mice [68]. Although the treated mice showed only a slight delay in motor neuron loss and slowing of motor performance decline, astogliosis and microgliosis were consistently attenuated in recipient mice in comparison to the controls [68]. Two weeks post-transplantation, transplanted cells were found mostly concentrated on lumbar, thoracic and cervical meninges, but considerable numbers of bisbenzimide positive cells were found in proximity to motor neurons within the spinal cord [68]. It is noteworthy that the use of bisbenzimide as a marker for cell transplantation has been questioned since the dye may transfer from labelled cells to host cells [69].

### Intraparenchymal delivery

Other authors have attempted to inject human or mouse MSCs directly into the dorsal horn of spinal cord of SOD1^G93A^ mice [70, 71]. In the Vercelli group experiments, hMSCs were found to engraft, migrate to the ventral horn close to α-motor neurons and survive more than 10 weeks after surgery, although without signs of differentiation into neurons or astrocytes [70]. Also, male but not female recipient mice showed a consistent (38%) increased motor neuron survival as well as reduced gliosis and attenuated astrocyte activation in comparison to control mice [70]. Though the authors claimed an extended lifespan in male treated mice, not all of the experimental groups were followed until the disease end-point, and a Kaplan-Meier survival curve was not shown.

### Intramuscular delivery

Since early pathologic mechanisms of disease involve destruction of neuromuscular junctions before motor neuron death, intramuscular injection of MSCs at early stages of disease has also been proposed. hBM-MSCs, genetically engineered to express green fluorescent protein (GFP) and to constitutively secrete GDNF, were injected into the forelimb triceps brachii muscles of pre-symptomatic SOD1^G93A^ rats [72].

The intramuscular transplantation of MSCs did not delay disease onset, however, survival was prolonged by 18 days. Furthermore, a reduction of endplate denervation in SOD1^G93A^ rats was observed, when compared to the control vehicle group [72]. However, MSCs were not able to regenerate motor end-plates, nor to reduce neuroinflammation [72]. Moreover, to obtain significant survival and integration of MSCs in the host, the induction of focal muscle injuries was necessary.

## MSCs in ALS: Clinical trials

### Mazzini trials: Intraparenchymal delivery of autologous BM-MSC

Despite the absence of any preclinical data, in 2001 Mazzini et al. embarked upon the first clinical trial in order to evaluate the safety and feasibility of MSC injection into the spinal cord of sporadic ALS patients [73]. The study comprised the recruitment of 7 SALS patients with spinal onset and severe lower limb impairment without respiratory complications [73]. Autologous mesenchymal bone marrow-derived stem cells were expanded for 3–4 weeks under good manufacturing practice (GMP) conditions and cytogenetic analysis, viability and cytofluorimetric analysis for characterization of antigens were carried out before infusion [73]. However, no detailed data was provided.

In this study, following three hours in serum-free medium, a variable number of cells ranging from 7 to 152 million were suspended in 1–2 ml of autologous CSF and transplanted directly into the parenchyma of spinal cord at thoracic level (T7-T9) [73, 74].

The patient follow-up was performed every 3 months for six years. After surgery, no signs of increased neurological defects or toxicity were observed, indicating that implantation into the spinal cord of ex vivo expanded MSCs was safe and well tolerated by patients [73, 74].

To further validate the safety of the therapy, in 2010 a second Phase I clinical trial was conducted in another 10 patients following the same protocol as described above, with slight modifications [75]. Cells (11–122 million) were transplanted in the anterior horn at the thoracic level (T4-T6) with different numbers of injection sites (2 to 5) [75]. Two years post-surgery, no tumour formation, side effects or toxicity had been detected [75].

A long-term (9 years) safety study, concluded in 2012, was carried out by the same authors on the basis of 19 ALS patients who underwent autologous MSCs implantation in two separate phase I studies from 2001 to 2003 [76]. Importantly, magnetic resonance imaging (MRI) analysis demonstrated no tumour formation or abnormal cell growth, indicating that ex vivo expansion of ALS patient-derived MSCs does not affect karyotype or cellular senescence, making their use safe in the clinic [76]. No data describing stem cell characterization were shown and evidence of engraftment in post-mortem tissues have not yet been described.

### Blanquer trial: Intraparenchymal delivery of autologous BMNC

Based on promising results in a mouse model of MND [77], Blanquer et al. tested the safety and tolerability of intraspinal transplantation of mononuclear cells derived from autologous bone marrow (BMNC) in ALS patients in an open label phase I clinical trial [78]. The study was performed on 11 sporadic ALS patients with spinal onset and no evidence of significant respiratory dysfunction [78].

Briefly, patients received two infusions of BMNC diluted in 1 ml of saline solution into two different sites along the posterior column of spinal cord at thoracic level after laminectomy, with the help of a specially designed micromanipulator [79]. A variable number (138,000 to 60,287 × 10^6^) of BMNC cells, including an average of 2.77 × 10^6^ CD34+, 2.31 × 10^6^ CD117+ and 1.30 × 10^6^ CD133+ cells, were injected [78].

After 1 year of follow up, no severe adverse effects or acceleration of the disease progression related to the treatment were observed, demonstrating the safety and feasibly of the described procedure in ALS patients [78]. Moreover, spinal cord pathology on post mortem material showed preservation of motor neurons at the injection sites [78]. Interestingly, preserved motor neurons were surrounded by spherical CD90+ cells negative for neuronal markers, CD45 (hematopoietic stem cells) and CD68 (monocytes/macrophages) markers [78]. These motor neurons did not show any sign of degeneration [77, 78].

### Karussis trial: Intrathecal and intravenous delivery of autologous MSCs

In 2010, a phase 1/2 clinical trial was conducted in Israel in 15 patients with multiple sclerosis (MS) and 19 ALS patients [80]. In order to enhance the potential benefits of MSC transplantation, after 40–60 days in culture, a median of 60 million autologous MSCs were transplanted intrathecally in combination with a mean of 20 million MSCs delivered intravenously [80]. Also, in 9 patients MSCs were pre-labelled with ferumoxides in order to track their fate in vivo by MRI [80].

At the end of 25 months of follow-up, no severe adverse effects were registered in any of the patients, with signs of disease stabilization in some patients during the first 6 months after the intervention [80]. Furthermore, MRI screening 24 h, 48 h, 1 and 3 months after the infusion of cells, showed the presence of ferumoxides in nerve roots, meninges and the parenchyma of the spinal cord. However, these results are not conclusive of the presence of MSCs in the CNS, since the contrast agent can be ingested by phagocytes which have migrated to inflammatory lesions [80]. Also, flow cytometry analysis and subsequent proliferative response assay of peripheral blood monocytes obtained from ALS patients 4 h and 24 h after infusion of MSCs, showed a dramatic increase in CD4+ CD25+ regulatory T cells, coupled with a reduction in activated dendritic cells and lymphocyte proliferation, demonstrating the immediate immunomodulatory properties of MSCs following transplantation [80].

### Oh trial: Repeated intrathecal infusion of autologous BM-MSCs

Very recently, a single open-label clinical trial was performed in order to evaluate the clinical feasibility and safety of two repeated infusions of autologous MSCs into the CSF by lumbar puncture in 8 ALS patients [81]. At intervals of 26 days, 1 × 10^6^ MSC cells per kg were injected diluted in autologous CSF [81].

After 1 year, no deaths were recorded and the procedure was considered safe, without long-term adverse effects [81]. In addition, CSF samples of two patients were collected before both the first and the second injection in order to evaluate cytokine levels. Il-10, TGF-β (I, II and III) and IL-6 levels were increased after MSC transplantation, while the level of the monocyte chemoattractant protein 1 (MCP-1) was decreased [81]. A phase II clinical trial in 64 ALS patients is on-going, with a placebo control group allowing proper evaluation of efficacy and safety (NCT01363401).

The same group performed repeated intrathecal MSC injections in another 37 patients between 2007 and 2010, with the aim of identifying MSC markers capable of predicting the response in ALS patients [82]. First, they measured the level of various trophic factors in MSC cultures from each patient by ELISA assay. They then transplanted MSCs from different patients in the SOD1^G93A^ mouse model and analysed differences in onset, MN loss and immunoreactivity [82]. The authors concluded that the beneficial effects (symptom improvement and slowing of decline) observed in a proportion of patients, were positively correlated with the increased capacity of MSC cells to secrete VEGF, ANG and TGF-β in vitro [82]. Moreover, the clinical efficacy observed in some patients was confirmed also in SOD1^G93A^ mice which exhibited prolonged survival and lower levels of neuroinflammation [82].

## Adipose tissue: A fat tissue source for mesenchymal stem cells

Mesenchymal stem cells represent an ideal source of adult stem cells for cell therapy given their immunosuppressive nature, low potential for immunogenicity and trans-differentiation capacity. However, the collection of MSC from bone marrow is an invasive procedure which can be painful and anaesthesia is often required [83]. Moreover, the proportion of stem cells within the total cell population in bone marrow aspirate is usually about 0.001–0.002% which leads to extended culture times and increased expense in order to obtain sufficient GMP cells for clinical application [83].

Subcutaneous (buttocks and abdomen) and visceral (omentum) white adipose tissue (WAT) may represent an alternative source of stromal adult stem cells since they can be obtained by minimally invasive, simple procedures such as liposuction or lipectomy and they are relatively abundant, representing 1% of WAT cells after processing [83–85]. Several names and abbreviations have been used to refer to adipose-derived mesenchymal stem cells. Here, the abbreviation ADSC will be used.

**Fig. 2:**
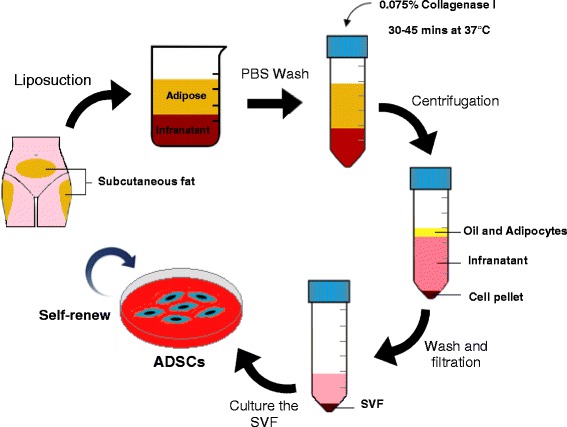
Isolation process to obtain ADSCs from human lipoaspirate. Fresh lipoaspirate is extensively washed in PBS to remove blood and contaminants. The adipose tissue is then enzymatically digested and the stromal vascular fraction (SVF) is obtained by filtration and centrifugation. Culture of the SVF in standard plastic tissue culture flasks results in the selection and expansion of the adipose stem cell population

In the literature, the method for isolation of ADSCs from human fat is performed following almost always the same protocol described by Zuk and colleagues [83]. An illustration of the ADSC isolation method is showed in Fig. 2.

Briefly, fresh lipoaspirate is washed extensively to eliminate red blood cells and contaminant debris, followed by extracellular matrix digestion with collagenase. After enzymatic neutralization, the homogenate is centrifuged to obtain the stromal-vascular fraction (SVF) pellet. The pellet is then suspended in culture medium and left overnight in a flask in the incubator at 37 °C with 5% CO_2_. After incubation, cells are washed with phosphate buffered saline (PBS) to remove residual non-adherent cells. The culture medium usually consists of Dulbecco’s modified eagle medium (DMEM) supplemented with foetal bovine serum (FBS), penicillin and streptomycin.

In spite of some differences in the expression of cluster of differentiation (CD) markers such as CD49d and CD106, ADSC cells are phenotypically similar to BM-MSC cells, showing fibroblast-like morphology, characteristic expression of mesenchymal stem cell markers (CD44^+^, CD105^+^, CD73^+^, CD90^+^, CD29^+^, CD45^−^, CD34^−^, CD14^−^ CD19^−^), lack of major histocompatibility complex class II (MHC-II) and the capacity to differentiate into osteoblasts, chondrocytes and adipocytes in specific culture conditions [83]. Thus, hADSCs comply with all the minimum criteria for the characterization of hMSCs, established in 2005, by the International Society for Cellular Therapy (ISCT) [86].

Like MSCs obtained from bone marrow aspirates, hADSCs secrete several soluble factors such as BDNF, NGF, hepatocyte growth factor (HGF), vascular endothelial growth factor (VEGF), insulin-like growth factor-1 (IGF-1) and basic fibroblast growth factor (bFGF) that may contribute to neurotropism, neuroprotection and tissue regeneration by paracrine mechanisms [87].

Even though the literature appears controversial, there is evidence supporting the potential of ADSCs to trans-differentiate into progenitors or mature cells of ectodermal origin, therefore opening the way to a future investigation of the feasibility of cell replacement in neurodegenerative disorders (Safford et al., 2002; Krampera et al., 2007; Ahmadi et al., 2012; Feng et al., 2014).

### In vitro neuronal differentiation capacity of ADSC

The capacity of ADSCs to differentiate in vitro into mature, stable and functional neurons is an open field of debate among researchers. Table 2 summarises the protocols for neuronal differentiation of ADSCs published in literature. Usually, the differentiation protocol consists of expanding ADSCs as adherent cells for several passages, followed by induction with media containing different cocktails of chemical agents, cytokines and growth factors [88–92].

**Table 2:** Summary of protocols to obtain neural trans-differentiation of hADSCs

Culture method	Differentiation protocol	Neuronal/glial marker expressed	Comments	Reference
Adherent method	24 h pre-incubation in DMEM/FCS 20% followed by 24 h incubation in DMEM containing BHA, valproic acid, forskolin, hydrocortisone and insulin.	Nestin (developing nervous system cells) NeuN (neuronal nuclei) GFAP (mature astrocytes) I-NFM (Neurons)	Cells started to lose their neuronal morphology after 4–5 days and all died in 14 days.	[88]
Adherent method	14 days incubation in DMEM containing insulin, indomethacin and IBMX	Vimentin (Schwann cells) Trk-A (central nervous system) NSE (early neuronal progenitors)	Low neuronal marker expression also in undifferentiated ADSCs. 25% differentiation rate obtained. Cells not able to generate action potentials.	[90]
Adherent method with or without human Schwann cells	24 h pre-incubation in DMEM/FBS 20% and β-mercaptoethanol followed by 8-16 h incubation in DMEM containing DMSO, β-mercaptoethanol and BHA	GFPA S-100 (Astrocytes and Schwann cells) NeuN Nestin Gal-C (Oligodendrocyte)	Cell morphology turned back to fibroblast shape after 72 h in normal basal medium. Co-culture with human irradiated Schwann cells enhanced survival (12 days) and expression of myelin proteins.	[91]
Adherent method	7 days incubation in DMEM containing bFGF followed by 7 days incubation in DMEM containing forskolin	GFPA I-NFM Tuj −1 (neuron specific β-III tubulin) Nestin SNAP-25 (synaptic marker) CNPase (Oligodendrocyte)	Inward and outward ion current in patch-clamp experiment. High mRNA expression of ion channels. Low differentiation marker expression also in undifferentiated ADSCs.	[92]
Neurosphere method followed by maturation on poly-D-lysine	8 days pre-incubation in DMEM containing β-mercaptoethanol and b-FGF followed by 7 days incubation in neural basal medium (N2B27) and further 7 days in N2B27 medium containing EGF and bGFG. Final maturation achieved from neurosphere dissociated progenitors after 14 day culture in N2B27 media containing retinoic acid and BDNF	MAP2 (mature neurons) Nestin Sox1(neural tube development) Pax6 (human neuroepithelium) Vimentin	Inward and outward ion currents in patch-clamp experiment. High mRNA expression of ion channels	[95]

*DMEM* Dulbecco’s modified eagle medium, *FCS* foetal calf serum, *BHA* butylated hydroxyanisole, *GFAP* glial fibrillary acid protein, *I-NFM* intermediate neurofilament, *IBMX* isobutylmethylxantine, *Trk-A* tropomyosin receptor kinase A, *NSE* neuron specific enolase, *DMSO* dimethyl sulfoxide, *S-100* S protein 100, *Gal-C* galactosylceramidase, *bFGF* basic fibroblast growth factor, *CNPase* 2^′^,3^′^-Cyclic-nucleotide 3^′^-phosphodiesterase, *EGF* epidermal growth factor, *BDNF* brain derived neurotrophic factor, *MAP2* microtubule-associated protein 2

A different method to obtain neuron-like cells from ADSCs is based on neurosphere formation capacity, generally achieved by culturing cells in EGF and bFGF conditioned serum-free media [93, 94]. A neurosphere is a cluster of cells containing neural precursors able to proliferate and survive as floating and non-adherent structures. The neurosphere can be dissociated, seeded onto a poly-L-lysine feeder layer and differentiated by adding neuronal induction components [93–95]. Alternatively, ADSCs are cultured in the presence of ESCs or Schwann cells either in the presence or absence of induction factors [91, 96]. In particular, when co-cultured with Schwann cells, ADSCs showed long-lasting (12 days) Schwann-like cell morphology and expression of myelin protein [91]. Pre-irradiation of Schwann cells excluded eventual cellular fusion artefacts [91]. Since the addition of supernatant from Schwann cell cultures failed to differentiate ADSCs, the authors speculated that the addition of specific chemical components or growth factors to the ADSC cultures, may promote the initial steps toward differentiation in vitro, which can be completed in the appropriate microenvironment and by required cell-to-cell interaction with mature cells in vivo.

The trans-differentiation of ADSCs towards motor neuron-like cells has also been reported. In 2012, Abdanipour and Tirahini [97] induced rat ADSCs to trans-differentiate into motor neuron-like cells by a two-step protocol. ADSCs were committed to neural progenitors expressing nestin, neurofilament 68 and neuro D by pre-induction for 24 h with selegiline. Selegiline is an inhibitor of monoamine oxidase B (MAO-B) which was used to trans-differentiate BM-MSCs into dopaminergic neural-like cells and proved to be a safer pre-inducer in comparison to the toxic β-mercaptoethanol and butylated hydroxyanisole compounds, widely used for ectodermal differentiation of MSC [97]. The maturation of the pre-induced ADSCs into a motor-neuron phenotype was then achieved by incubation with Shh and RA, resulting in a differentiation efficiency of around 70%. The resultant motor neuron-like cells (MNLCs) were characterized by an initial and transient high expression of oligo-2 and islet-1 (markers for early motor neuron commitment during in vitro differentiation), followed by a decrease in these two markers accompanied by an increase in the expression of the motor neuron marker HLBX9 [97]. Mature MNLCs, but not high HLBX9 expressing cells, were functionally tested for the capacity to release pre-synaptic vesicles by staining and destaining with the fluorescent probe FM1–43. Quantitative analysis of vesicle release after stimulation, showed a 4-fold increase in comparison to pre-induced ADSCs. MNLCs, also formed innervation-like contacts with myotubes in a co-culture in vitro system [97].

Very recently, the same authors proposed a different method to obtain MNLCs from rat ADSCs [98]. ADSCs were first converted into neurospheres by induction with NM medium consisting of DMEM, B27, EGF and bFGF for 7 days. Next, neural stem cells (NSC) were obtained by neurosphere dissociation into single cells, and incubation for 10 days with NM supplemented with 10% FBS. Maturation into motor neurons was achieved by incubation with Shh and RA for 5 days, followed by addition in culture media of BDNF, GDNF, CTNF and NT-3 for another 7 days [98]. During the maturation process of ADSC-NSCs into motor neurons, an increase in the expression of islet-1, HB9 and ChAT was observed, with MN-like cells positive for the neural marker MAP2 at day 14 of maturation. These findings were documented by both immunocytochemistry and RT-PCR. The functional activity of mature MNLCs was investigated by quantification of the release of synaptic vesicles by FM1–43 loading and release experiments upon ion stimulation. The synaptic vesicle activity correlated with changes in intracellular calcium concentration and membrane depolarization, as demonstrated by further investigations utilizing calcium and voltage-sensitive dyes [98].

### Is the trans-differentiation of ADSCs into neurons real?

The differentiation into neurons is commonly described as morphological changes such as body retraction, bi- or multi-polar shape and branching extension, and by the expression of neuronal markers revealed by immunohistochemistry (IHC), western blotting (WB) and quantitative RT-PCR analysis. However, several studies showed that the neural-like phenotype obtained by chemical induction was a very fast but transient event which may be a result of cytoskeletal rearrangement due to the toxicity of compounds added into the induction media [99, 100]. Furthermore, several neuronal markers considered to confirm neuron maturation such as Nestin, NSE, trk-A and vimentin are already present at low levels in undifferentiated ADSCs [89, 90, 92, 95, 101].

Thus, morphological changes and expression of neuronal markers on the cell surface should not be considered as a definitive proof of neuronal differentiation, and functional characterization (i.e. electrophysiology) is necessary. However, very few studies confirmed electrical activity of ADSC-derived neurons by patch-clamp experiments [92, 95].

In addition, the efficiency and reproducibility of differentiation protocols described in the literature is low, and neuronal induction of ADSCs gives rise to a heterogeneous population of undifferentiated cells, neuron-like cells and astroglial-like cells with different degrees of maturation [91, 92]. Together with the fact that untreated ADSCs often show slight expression of neuronal progenitor markers, these results suggest the presence of specific cell subtypes with different neuronal differentiation potential [102]. This is supported by the existence of variability in the secretome of hADSCs obtained from different donors and maintained in separated cultures [103].

### In vivo studies with ADSCs

For the first time in 2013, the therapeutic potential of ADSCs was investigated in the SOD1^G93A^ mouse model [104]. ADSCs were isolated from C57BL/6 GFP-expressing mice, and a total of two million cells were injected into the tail vein of B6SJL-SOD1^G93A^ mice after the first clinical signs of disease. The control group received injection of PBS only as vehicle. mADSCs delayed the motor performance decline and transiently attenuated motor neuron death, in comparison to the controls. However, no differences in the degree of astrogliosis, nor in survival were observed between the two groups [104]. The authors claimed that GFP-positive cells were found to migrate into damaged CNS areas, such as the grey matter in the spinal cord, however, low magnification images showing the entire spinal cord section or co-staining with neuronal specific markers were not shown and histology on PBS-treated control spinal cord sections was not performed. Finally, increased levels of trophic factors such as GDNF and bFGF were found in spinal cord homogenates of ADSC injected mice. This was despite the fact that mADSC used in this study were not capable of producing GDNF in vitro. It is likely that, in addition to trophic factor production and secretion, ADSCs could have an indirect biological effect by stimulating the secretome of surrounding astrocytes, which are known to produce GDNF [104].

Recently, another group tested the therapeutic potential of human ADSCs isolated from three healthy donors aged 64, 69 and 84 [105]. Female transgenic SOD1^G93A^ mice before clinical evidence of disease received 1 × 10^6^ hADSCs by intravenous (IV) or 2 × 10^5^ hADSCs by intracerebroventricular (ICV) injections. As a control, the sham group received PBS only.

Rotarod test and paw grip endurance were monitored and a reduction of 15% was considered as disease onset. The endpoint (survival) was defined by the lack of a righting reflex within 30 s after being placed on their side [105]. The authors found that disease onset in mice that received ICV injections was significantly delayed by 26 days, while in mice infused IV there was only an 11 day delay in disease onset. Remarkably, survival was prolonged by 24 days and 9 days respectively in ICV and IV mice when compared to the controls [105].

IHC evaluation of the spinal cord of ICV transplanted mice revealed that 6.8% of transplanted cells survived up to 4 weeks post-injection. However, only 0.7% migrated to the grey matter of the lumbar spinal cord and very few cells were positive for neuronal markers (MAP2 or I-NFM). In contrast, after IV infusion very few undifferentiated cells were found to engraft in the meninges of the spinal cord [105]. In addition, when the TUNEL assay was performed on spinal cord from ICV mice, reduced levels of apoptosis were seen in the anterior grey matter compared to IV and control recipients. Also, RT-PCR and ELISA assays on spinal cord homogenates showed a significantly higher concentration of neurotrophic factors in the ICV group in comparison to controls [105]. ELISA assay on the supernatant from hADSC cultures showed high levels of neurotrophic factors that are relevant in neuroprotection such as IGF-1 and VEGF [105]. The anti-apoptotic effect was further confirmed in vitro by culturing primary neural cells from normal mice with hADSC conditioned media [105].

To date, these are the only two published studies using ADSCs in an experimental model of ALS. However, the therapeutic potential of ADSCs has been tested in other experimental models of neurodegeneration. In particular, the use of ADSCs showed encouraging results in animal models of chronic stroke, Parkinson’s disease, Alzheimer’s disease and traumatic brain injury and ageing [106–109]. The use of ADSCs in ALS patients has not been tested so far. However, currently two different clinical trials are recruiting participants.

A clinical trial sponsored by The Royan Institute in Iran will test the safety of intravenous injections of ADSCs derived from healthy donors (2 million cells/kg) in 8 ALS patients (ClinicalTrial.gov identifier: NCT02492516). The Andalusian Initiative for Advanced Therapies is recruiting 40 ALS patients for a phase I/II, multicentre, randomized, placebo controlled clinical trial to evaluate the safety and efficacy of intravenous infusion of different concentrations of autologous ADSCs (ClinicalTrials.gov identifier: NCT02290886).

### MSCs for ALS therapy: Proposed mechanisms of action

The in vitro differentiation of mesenchymal stem cells into neuron-like cells brought great enthusiasm into the idea of cellular replacement as a therapeutic strategy in ALS. However, there are many practical issues to be solved. The real trans-differentiation of MSCs into neurons has been questioned and further investigation in order to study molecular pathways and optimized protocols enhancing the efficiency, stability and degree of maturation are needed. Also, only a few studies have reported MSCs or MSC-derived NSCs showing signs of in vivo maturation when transplanted into mice (detailed in the next section).

More importantly, for therapeutic efficacy, the transplanted cells should engraft, migrate to affected areas of degeneration, survive, mature and integrate into the pre-existing neuronal circuits forming synapses, extending long axon projections to reach muscles and regenerating neuromuscular junctions. All this must occur within a hostile microenvironment where other motor neurons are dying and activated microglia and astrocytes are sustaining an inflammatory milieu [30, 31].

Moreover, different pathological mechanisms described in ALS such as glutamate excitotoxicity [22], oxidative stress [26] and loss of metabolic support [110] may affect the viability of transplanted cells and inhibit maturation. Finally, stem cell maturation and integration into the host neuronal circuits could last a relatively long time, which may not be compatible with the fast progression rate of the disease.

Besides the challenge of cell replacement in ALS, in recent years increased attention has been paid to the “bystander effect” mechanism through which MSCs could exert their therapeutic effect. Different mechanisms have been proposed to explain the role of MSCs in neuroprotection. Although the precise mechanisms are still unknown, the secretion of anti-inflammatory cytokines and growth factors by MSCs may influence the progression of ALS in multiple ways, including endogenous regenerative processes such as neuronal plasticity, angiogenesis and axonal re-myelination [66, 111].

The delivery of growth factors such as BDNF, insulin-like growth factor-1 (IGF-I), vascular endothelial growth factor (VEGF) and glial derived neurotrophic factor (GDNF) into ALS experimental models has been very promising since these factors were shown to be neuroprotective, improved motor function and prolonged motor neuron survival [112]. However, translation into the clinic failed to provide any beneficial effect in ALS patients [112]. This is thought to be related to the small amount of growth factor that effectively reached the central nervous system (CNS) either because of an inability to cross the blood brain barrier or because of the short half-life after intravenous injection [112]. Moreover, intrathecal injections of growth factors did not result in relevant benefits for ALS patient [112].

The use of MSCs as “carriers” for the uninterrupted supply of growth factors has been proposed, having shown positive results in an ALS rodent model [113, 114]. Indeed, MSCs transduced to overexpress specific growth factors (GDNF, VEGF) or neuroprotective agents (e.g. glucagon-like peptide 1) transplanted into SOD1^G93A^ rodent models, significantly preserved neuromuscular junctions, attenuated motor neuron death, improved motor function, delayed symptom onset and prolonged survival [113, 114]. Recently, the Karussis group and Brainstorm-Cell therapeutics completed an open lab phase 1/2 clinical trial evaluating the safety and feasibility of intrathecal injections of MSCs induced to secrete neurotrophic factors (BDNF, GDNF) (NurOwn™) in 12 ALS patients (NCT01051882). Although, no results have been published so far, the details of another two separate active clinical trials can be found at ClinicalTrials.gov. An open label phase 2a escalating-dose trial (NCT01777646) and a phase 2 randomized, double-blind trial (NCT02017912) in order to evaluate safety and efficacy of multiple intramuscular and intrathecal combined injection of NurOwn™ cells into ALS patients are reported as in progress.

Through paracrine activity and cell-to-cell contacts, MSCs were able to induce astrocytes and glial cells to secrete enhanced levels of GDNF, VEGF and CNTF (ciliary neurotrophic factor) both in vitro and in the SOD1^G93A^ mice, resulting in anti-apoptotic effects and motor neuron protection [104, 115].

Another potential mechanism by which MSC could participate in tissue repair, is in the secretion of exosomes [116]. Exosomes are membrane vesicles originating from the inner cell membrane, which contain proteins, mRNAs and microRNAs. The content of exosomes, after their secretion from stem cells, could be transferred to neighbouring cells and mediate a plethora of biological pathways from free radical scavenging to the activation of self-regenerative programmes [117]. Interestingly, exosomes derived from murine adipose derived stem cells (ADSCs), were able to protect both naïve and SOD1^G93A^ transfected (overexpression of the human SOD1 gene with the G93A mutation) NSC34 motor neuron-like cells from oxidative stress in an in vitro culture system, with a significant reduction of apoptosis events and an increase in cell viability [118].

MSCs may act by modulating astrocyte dysfunction since they showed the ability to induce expression of glutamate re-uptake transporter 1 (GLT1) and inhibit caspase-3 cleavage in SOD1^G93A^ mutated astrocytes, thus limiting excitotoxicity [119].

Several studies on experimental models of ALS demonstrated the capacity of MSCs to attenuate astrogliosis [67, 68, 70]. When transplanted into SOD1^G93A^ mice, MSCs may exert immunomodulatory effects indirectly by stimulating host cells to secrete anti-inflammatory interleukins (IL) such as IL-10, IL-3 and IL-13 [68].

Intrathecal delivery of hMSCs into SOD1^G93A^ mice slowed disease progression, but increased lymphocyte infiltration into the spinal cord [120]. However, hMSCs cultured with peripheral blood mononuclear cells (PBMC) derived from ALS patients increased the proportion of regulatory T cells accompanied by enhanced production of anti-inflammatory cytokines such as IL-4, IL-10 and transforming growth factor-beta (TGFβ) [120]. Thus, when injected into the cerebrospinal fluid (CSF) of ALS mice, MSCs could exert their beneficial immunomodulation by stimulating regulatory T cell proliferation, activation and migration to areas of CNS inflammation [120]. MSCs were shown to inhibit maturation and activation of dendritic cells in vitro, and prevent lymphocyte migration into the spinal cord of experimental autoimmune encephalomyelitis mice [121, 122]. MSCs were also able to suppress proliferation, maturation and activation of pro-inflammatory Th1 and Th17 cells, with a concomitant switch toward active CD4^+^ CD25 Foxp3 regulatory T cells in vitro [123].

T regulatory cells are reduced in patients with aggressive ALS and reduced levels of these cells in early disease correlates with rapid progression of neurodegeneration [124]. Interestingly, peripheral blood monocytes obtained from ALS patients 4 h and 24 h after an intravenous infusion of MSCs, showed a dramatic increase in CD4+ CD25+ regulatory T cells, coupled with a reduction in activated dendritic cells and lymphocyte proliferation [80].

The establishment of a sustained pro-inflammatory milieu in ALS spinal cord has been demonstrated which is probably accompanied by the shift of microglia cells from an anti-inflammatory state (often referred to as M2 in the literature) to an active neurotoxic state (often referred to as M1 in the literature) [125].

Of interest, MSC-conditioned media significantly inhibited the production and secretion of pro-inflammatory cytokines in microglia activated by lipopolysaccharide (LPS) [126] [127]. This, has been attributed to the capacity of MSCs to secrete TGF-β, which in turn inhibited the NFκB pathway and restored a protective microglial phenotype [127]. Thus, through paracrine effects, MSCs could modulate the functional properties of microglia by switching the detrimental M1 activated microglial state to the beneficial M2 activated microglial state after LPS induction [127].

Therefore, the immunomodulatory properties of MSCs may play an important role in attenuating neuroinflammatory processes during the progression of ALS and further work is needed in order to explore this specific mechanism.

**Fig. 3:**
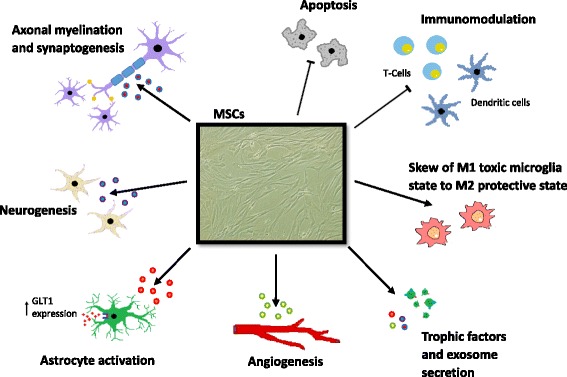
Potential mechanisms of mesenchymal stem cell efficacy in neurodegeneration. Transplanted MSCs may provide therapeutic responses through paracrine effects and cell-to-cell contacts with resident neural cells. The capacity of MSCs to secrete cytokines, growth factors and exosomes could potentially induce and support regeneration processes, including angiogenesis, synaptogenesis, axonal re-myelination and neurogenesis. Because of their immunomodulatory properties, MSCs could attenuate inflammatory responses in the central nervous system by inhibiting maturation and migration of dendritic cells, suppression of lymphocyte activation and proliferation, and by reducing gliosis. Moreover, MSCs possess anti-apoptotic properties, and may limit excitotoxicity by modulating astrocyte functions

An overview of the potential beneficial effects that MSCs could exert in modulating pathophysiology in neurodegenerative conditions is summarized in Fig. 3.

### Delivery route for MSCs in ALS

One of the major technical issues in ALS cellular therapy is to effectively deliver stem cells into the CNS. Several studies demonstrated that injecting human stem cells directly into the spinal cord of ALS rodents is feasible, safe and efficient [40, 70, 71, 128]. The surgical procedure for intraspinal infusion of hMSCs into the spinal cord of ALS patients by specifically designed microinjection manipulators have also been shown to be safe in open-label clinical trials [75, 76, 78, 79, 129]. However, direct CNS injection remains an invasive procedure which could cause serious clinical complications and permanent damage to CNS areas already affected by the disease. Thus, it might be a considerable ethical issue in clinical trials when investigating the efficacy of the therapy, especially where placebo controls are required.

Since early pathologic mechanisms of disease involve destruction of neuromuscular junctions before motor neuron death, intramuscular injection of MSCs at an early stage of disease has also been proposed. Intramuscular injections of hMSCs engineered to overexpress GDNF into SOD1^G93A^ rats resulted in delayed disease onset and prolonged survival, but MSCs were not able to regenerate motor end-plates, nor to ameliorate motor neuron loss [72].

Given the immunomodulatory properties, together with the homing capacity in response to inflammatory signals, hMSCs showed beneficial effects when infused intravenously in ALS animal models [58, 61]. However, even though intravenous infusion could be the easiest and safest way for stem cell delivery, very few cells appear to be able to migrate, penetrate and engraft into the spinal cord parenchyma. Thus, a large number of cells may need to be transplanted in order to obtain effective results in human subjects.

**Fig. 4:**
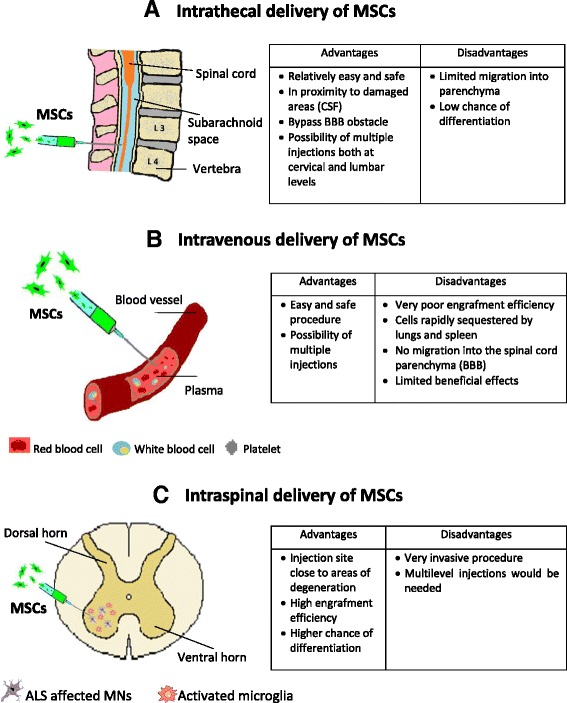
Delivery strategies for the transplantation of MSCs in ALS. **a** Intrathecal delivery of MSCs into the spinal cord CSF; **b** Systemic delivery of MSCs; **c** Local delivery of MSCs directly into the spinal cord parenchyma. For each delivery route, advantages and disadvantages are summarized

The delivery of stem cells into the CSF surrounding the spinal cord by intrathecal injection may be a useful compromise, since stem cells would be placed in proximity to damaged areas without direct delivery into the spinal cord parenchyma. Furthermore, it would be possible to perform multiple injections both at the cervical and lumbar levels, allowing stem cells to migrate towards more affected areas of neurodegeneration/inflammation [67]. In some preclinical studies, stem cells were able to migrate from the CSF into the parenchyma, however, the mechanisms of migration remains unknown [64, 68]. Figure 4 is a representation of different proposed strategies to deliver MSCs into ALS patients with the relative advantages and disadvantages.

### Strategies to enhance tissue penetration

The study of CNS inflammation models allowed the discovery of chemokines, receptors, adhesion molecules, and inhibitors that govern and regulate lymphocyte migration, extravasation and penetration into the CNS parenchyma. Even though the migration of lymphocytes from the blood to the CSF has been extensively studied, very little is known about the transmigration of lymphocytes from the perivascular space to the CNS parenchyma, where astrocytes and the basal membrane constitute a second impermeable barrier [130].

Curiously, important chemokines released during inflammation which stimulate lymphocyte migration through the BBB such as CXCL12 and CXCL10, were shown to inhibit infiltration into the parenchyma, inducing lymphocytes to persist in the perivascular space [131, 132]. Conversely, production of metalloproteases such as MP-2 and MP-9 seems to be necessary in order to break the dystroglycan basal membrane required for penetration into the CNS [130, 133].

MSCs express several chemokine receptors and homing properties similar to that of lymphocytes which allow them to migrate into damaged or inflamed tissues. However, donor age and the number of passages in culture were shown to negatively influence the stem cell’s expression of homing factors [134].

Interestingly, Corti and colleagues have performed intrathecal and intravenous injection in SOD1^G93A^ mice of hiPSC-derived neural cells after being sorted for expression of the integrin VLA4 (CD49d) by FACS [49]. A considerable number of transplanted cells were able to migrate into the grey and white matter of spinal cord [49].

Engineering MSCs in order to induce overexpression of chemokine receptors or factors involved in homing and migration to sites of inflammation, may enhance the delivery into the spinal cord parenchyma. The characterization of chemokines and relative concentration in spinal cord and CSF of ALS models during different stages of disease would be of interest to identify the best candidate and also the peak of inflammatory chemokine production in order to define the ideal time of intervention for stem cell injection.

### Future directions in clinical trials

During the past two decades, several clinical trials investigated the use of stem cells in patients with ALS, mostly focusing on the safety and feasibility of the intervention. Nonetheless, as reported by Appel and Armon, stem cells have often been transplanted into ALS patients with limited preclinical data, without providing details on adverse effects and using small numbers of participants [135]. Moreover, the long-term safety of stem cell transplantation (e.g. non acceleration of disease progression associated with the cell implantation) has been evaluated by comparison with historical control groups. This is an important limitation since heterogeneity in the patient population and differences in clinical care between the treated group and historical controls may affect the readouts [135].

Although single-armed, small phase I/II clinical trials found that cell-based therapy for ALS is relatively safe and feasible, it is uncertain whether stem cell transplantation may be clinically beneficial leading to functional improvements and of the slowing of disease progression. The majority of the clinical trials were principally focused on the safety and feasibility of the surgical procedure related to the stem cell implantation. Secondly, these clinical trials were not powered to demonstrate any clinical benefit [136].

In 2016, Abdul et al. published a systematic review aiming to assess the effectiveness of stem cell therapy in people with ALS, compared with a placebo or no additional treatment [136]. However, the authors could not identify any randomized controlled trial (RCT), quasi-RCT or cluster RCT involving the use of stem cells in ALS/MND patients. Thus, there is an absence of high-quality published evidence to assess the safety and efficacy of stem cell transplantation in ALS [136]. Moreover, when analyzing the single arm phase I/II clinical studies available in the literature, the authors found substantial variability between clinical trials in terms of selection criteria, intervention methods and objective outcomes, with the involvement of small number of participants and a short-term follow-up period [136] .

To investigate the long-term safety and efficacy of cellular therapy for ALS, well-designed prospective randomized-controlled trials with larger sample size, long-term-follow up and standardization of cell products, are urgently needed [136].

Recently, the ALS Clinical Trials Workshop (Airlie Conference, Virginia, 2016), driven by the ALS community, clinicians, researches, industry representatives, government representatives, ALS patients and family members, released and submitted to the FDA the draft version of the “Guidance for Industry on Drug Development for Amyotrophic Lateral Sclerosis”. The manuscript has been drafted with the intention of improving and accelerating the drug development process, including guidelines for a more effective clinical trial design in ALS [137]. In particular, the guideline highlighted the importance of reducing clinical and genetic heterogeneity when establishing the patient selection criteria. With regards to clinical heterogeneity, inclusion and exclusion criteria for patient enrolment must be well justified for each study, which could vary depending on the phase of development [137].

Selection criteria should also depend on the specific trial goals, since some therapies may be more effective in early stage of disease or in specific sub-group of patients. Thus, investigators should endeavor to incorporate reliable predictive and prognostic biomarkers for clinical trial eligibility or stratification criteria [137].

Because of the multifactorial nature of ALS, post-hoc analysis of reliable biological markers and neurophysiological data is extremely important, since in specific sub-groups of patients a beneficial effect could be missed during the analysis. However, responder analyses must only be used as hypothesis generating, which will need to be confirmed and further investigated in future trials [137].

Importantly, although early phase trials are not meant to investigate efficacy, for cell-based therapies which embrace potentially invasive delivery methods and lifelong biological effects, evaluation of efficacy should be included in the design of phase I trials. Furthermore, a long-term monitoring plan to evaluate tumorigenesis, stem cell engraftment and long-term efficacy should be included [137].

Finally, RCTs with placebo controls is considered as the gold standard for clinical investigation. However, in rare, rapidly progressive and untreatable diseases such as ALS, the requirement for a placebo could be revisited, especially if the intervention involves invasive methods. While in phase III clinical trials a randomized placebo group is indispensable, in earlier phase studies, the use of historical controls and predictive algorithms could be accepted. However, the development of target-specific biomarkers, standardization of outcome measures and validation of surrogate end-points is essential, especially in cell-based studies in which the mechanisms mediating the therapeutic effect are not well established.

## Conclusions

During the last decade, the great advances achieved in regenerative medicine have created an unprecedented enthusiasm and new hope for amelioration of the devastating and until now incurable disease that is ALS. The initial exciting idea of replacing lost of motor neurons, with MSCs is unlikely to be achieved, although other strategies may provide some promise for stem cell derived motor neuron replacement [138]. Indeed, transplanted cells should differentiate and integrate with the host spinal motor circuits within the toxic and non-permissive environment that characterizes ALS. However, the neuroprotective and immunomodulatory potential of MSCSs could match perfectly with the multifactorial nature of ALS.

Among different types of stem cells, MSCs represent a promising candidate for clinical application. However, several technical issues need to be addressed including: route of administration, optimal dose, and differentiation state and neuroprotective mechanisms of transplanted cells. In addition, in the majority of pre-clinical experiments stem cell injection was performed before disease onset. This is an important limitation for translation into the clinic where the diagnosis takes several months and early markers of disease are lacking [139]. Another limitation is the exclusive use of the SOD1^G93A^ model, which may reflect the pathological features of only a very small proportion of patients. Although these mice represent a robust model of ALS, only a small proportion of ALS cases are caused by SOD1 mutations [140]. Thus, the use of other ALS models such those driven by mutant TDP-43 and C9ORF72 expansions would be of interest [141].

Also, despite the excellent utility of the SOD1^G93A^ mice in revealing pathological ALS features, translation of promising therapies from this model has failed in human clinical trials [142]. Apart from considerable differences between rodents and humans, the answer to this problem could be attributed to the existence of a high rate of intra- and inter-laboratory variability [142]. Such variability could be improved by a more meticulous preclinical design, use of defined inbred mouse strains, transgene copy number analysis and trying to standardize preclinical investigation methods [142, 143].

In general, the presence of injected stem cells within the host nervous system of treated rodents was evaluated only a few days or weeks post-transplantation. Thus, the design of experiments looking at the long-term survival and integration of cells is necessary.

Evaluation of the presence of injected cells in the host has been carried out by the exclusive use of immunohistochemistry. Adopting advanced microscopy techniques such as confocal and two-photon microscopy would be of great interest to confirm stem cell engraftment, which could also allow tracking in vivo the fate of transplanted cells [144, 145].

In addition, rate of proliferation, stemness properties, longevity and differentiation capacity of MSCs declines considerably with time when culturing cells as monolayers [146]. The advent of 3D culture systems, along with enormous progress in the fabrication of biomaterials must be considered. When maintained in 3D culture systems such as hanging drops, low-adhesion plates, porous scaffolds or hydrogels, hMSCs showed increased proliferation and migration capacity, enhanced colony-forming efficiency, higher expression of stem cell markers, greater neuronal differentiation ability and greater cellular engraftment after transplantation in animals [146–148].

Thus, the regenerative potential of stem cells could be considerably improved by adopting 3D culture methods. Transplantation of bio-scaffolds or encapsulating MSCs to sustain favorable conditions for stem cell survival, growth, migration and maturation is showing promising results in experimental models of spinal cord injury, traumatic brain injury and nerve regeneration, and must be considered in ALS models where the presence of a hostile microenvironment is one of the main factors that negatively affects stem cell engraftment and therefore therapeutic potential [147, 149, 150].

The optimal maturation level for the transplantation of stem cells to obtain the greatest therapeutic benefit is also unclear. If undifferentiated cells may represent the best way to obtain immunomodulation and trophic factor production, induced neural progenitors may overcome the possibility of tumour formation, along with the possibility of in vivo maturation and integration into existing neuronal circuits.

A combination strategy could be interesting, where intravenous infusion of immature cells may generate permissive environmental conditions for the successive implantation of neuronal committed cells within the CSF. This would require an in-depth study of the maturation process of MSCs into neurons, trying to understand, optimize and standardize differentiation protocols that promote the generation of stable precursors.

Last but not least, although autologous transplantation can reduce the probability of immune rejection, it has been reported that patient-derived MSCs may have impaired or reduced therapeutic effects [151, 152]. Other evidence indicates no functional alteration or accelerated cellular senescence in BM-MSCs derived from sporadic cases of ALS [153]. Moreover, it was shown that there was the possibility to functionally restore defective ALS-derived MSCs by correcting alterations in DNA methylation [153, 154]. However, the identification of MSC biological markers predictive of a positive/negative therapeutic response in ALS patients would be of great value [82]. In relation to the use of autologous MSCs in patients carrying ALS causative genetic mutations, stem cells could be genetically corrected by adopting CRISPR-Cas9-mediated gene editing technology [155].

In conclusion, several strategies have been tested in the SOD1^G93A^ mouse model, but with different hurdles. All the discussed parameters should be reconsidered and optimized before the translation of stem cell therapy from mice to humans in order to avoid undesirable delays or therapy failure as has happened for most of the promising results derived from SOD1^G93A^ transgenic mouse models, with failure of translation into clinical benefits for ALS patients.

## Abbreviations

ADSC: Adipose derived stem cells
ALS: Amyotrophic lateral sclerosis
BBB: Blood brain barrier
BDNF: Brain derived neurotrophicfFactor
bFGF: basic fibroblast growth factor
BM: Bone marrow
BMNC: Bone marrow mononuclear cells
CD: Cluster of differentiation
CNS: Central nervous system
CNTF: Ciliary neurotrophic factor
CSF: Cerebrospinal fluid
DCX: Doublecortin
DMEM: Dulbecco’s modified eagle medium
FALS: Familial amyotrophic lateral sclerosis
FBS: Foetal bovine serum
FUS: Fused in sarcoma protein
GDNF: Glial derived neurotrophic factor
GFAP: Glial fibrillary acidic protein
GFP: Green fluorescent protein
GLT1: Glutamate re-uptake transporter 1
GMP: Good manufacturing practice
hESC: human embryonic stem cells
HGF: Hepatocyte growth factor
ICV: Intracerebroventricular
IGF-I: Insulin-like growth factor-1
IHC: Immunohistochemistry
IL: Interleukin
iPSC: induced pluripotent stem cells
ISCT: International Society for Cellular Therapy
IV: Intravenous
MAO-B: Monoamine oxidase B
MCP-1: Monocyte chemoattractant protein 1
MHC-II: Major histocompatibility complex class II
MND: Motor neuron disease
MNLCs: Motor neuron-like cells
MNPs: Motor neuron progenitors
MNs: Motor neurons
MS: Multiple sclerosis
MSCs: Mesenchymal stem cells
NGF: Nerve growth factor
Ngn1: Neurogenin1
NPs: Neural progenitors
NSC: Neural stem cells
NT-3: Neurotrophin 3
OPTN: Optineurin
PBMC: Peripheral blood mononuclear cell
PBS: Phosphate buffered saline
PNN: Perineural net
RA: Retinoic acid
RCT: Randomized controlled trial
SALS: Sporadic amyotrophic lateral sclerosis
SCI: Spinal cord injury
Shh: Sonic hedgehog
SMA: Spinal muscular atrophy
SOD1: Superoxide dismutase 1
SVF: Stromal vascular fraction
TARDBP: TAR DNA-binding protein 43
TGFα: Transforming growth factor-alpha
TGFβ: Transforming growth factor-beta
VCP: Valosin containing protein
VEGF: Vascular endothelial growth factor
WAT: White adipose tissue
WB: Western blotting
WT: Wild-type
